# Metabolic Outcome of Female Mice Exposed to a Mixture of Low-Dose Pollutants in a Diet-Induced Obesity Model

**DOI:** 10.1371/journal.pone.0124015

**Published:** 2015-04-24

**Authors:** Danielle Naville, Emmanuel Labaronne, Nathalie Vega, Claudie Pinteur, Emmanuelle Canet-Soulas, Hubert Vidal, Brigitte Le Magueresse-Battistoni

**Affiliations:** CarMeN Laboratory, INSERM U1060, Lyon-1 University, INRA UMR1397, INSA-Lyon, Oullins, France; University of East Anglia, UNITED KINGDOM

## Abstract

Pollutants are suspected to contribute to the etiology of obesity and related metabolic disorders. Apart from occupational exposure which concerns a subset of chemicals, humans are mostly exposed to a large variety of chemicals, all life-long and at low doses. Food ingestion is a major route of exposure and it is suggested that pollutants have a worsened impact when combined with a high-fat diet. In the experimental studies described herein, we aimed to add further evidence on the metabolic impact of food pollutants using a recently set up model in which mice are life-long fed a high-fat/high-sucrose diet (HFSD) with/without common food pollutants shown to exhibit metabolic disrupting activities. Specifically, this mixture comprised bisphenol A, dioxin, polychlorobiphenyl PCB153, and phthalate and was added in HFSD at doses resulting in mice exposure at the Tolerable Daily Intake dose range for each pollutant. We herein focused on the 7-week-old females which exhibited early signs of obesity upon HFSD feeding. We observed no signs of toxicity and no additional weight gain following exposure to the mixture but alleviated HFSD-induced glucose intolerance in the absence of alteration of gluconeogenesis and steatosis. It suggested that the observed metabolic improvement was more likely due to effects on muscle and/or adipose tissues rather than on the liver. Consistently, female mice exhibited enhanced lean/fat mass ratio and skeletal muscle insulin sensitivity. Moreover, expression levels of inflammatory markers were reduced in adipose tissue at 7 but enhanced at 12 weeks of age in agreement with the inverse alterations of glucose tolerance observed at these ages upon pollutant exposure in the HFSD-fed females. Collectively, these data suggest apparent biphasic effects of pollutants upon HFSD feeding along with obesity development. These effects were not observed in males and may depend on interactions between diet and pollutants.

## Introduction

Diabetes and obesity are the major public health challenge of the 21^st^ century, with prevalence reaching epidemic proportions worldwide. Because of the exponential production and usage of synthetic chemicals that have paralleled the disease epidemic, it was hypothesized that pollutants could interact with physiological mechanisms regulating glucose and lipid metabolism [1]. The term of metabolic disruptors was next proposed [2] to encompass chemicals with endocrine disrupting activity and for which exposure has been linked to diabetes and obesity in epidemiologic studies [2–4] as well as in experimental studies [5–8].

Based on their resistance to biodegradation, synthetic chemicals are classified in persistent organic pollutants (POPs) and short-lived pollutants. POPs are widespread environmental pollutants that comprise intentionally manufactured products (e.g., the polychlorinated biphenyls, PCBs) or by-products of various industrial processes (e.g. dioxins). POPs are lipophilic and biomagnified up the food chain. Thus, diet is the main route of exposure [9, 10]. Non-persistent pollutants include, among others, bisphenols and phthalates. They are produced at high volumes and have numerous applications, notably in the plastics industry [11, 12]. Hence, they are found in daily consumables and measurable in urines of most individuals [13]. In addition, pollutants can transfer from mother to fetus through the placenta during pregnancy and through breast feeding [14]. Consequently, humans are exposed to a huge variety of potentially harmful pollutants, the overall impact of which cannot be predicted from the effects of each individual pollutant [15]. Yet, human health risk assessment is based on individual chemicals, with the determination of a Tolerable Daily Intake (TDI) [16]. Consequently, health risk assessment does not consider the mixture effect and dose addition may underestimate cumulative risk evaluation. To add to the complexity of the studies, some adverse effects for BPA occurred in rodents with doses lower than the TDI reference dose [17], and experimental studies showed that a high fat diet could amplify the adverse metabolic effects of BPA in rats [18] and PCB153 in mice [19].

In the present study, we set up an experimental model of exposure to a chemical mixture. Our originality stems from the use of a diet-induced obesity rodent model coupled with a lifelong exposure to pollutants covering the early vulnerable periods of life (gestation and lactation) [20], and an artificial mixture combining persistent (2,3,7,8-tetrachlorodibenzo-p-dioxine, TCDD and PCB153) and non-persistent pollutants (BPA and diethylhexylphthalate, DEHP) in the TDI range. These pollutants could be found in a Western-diet and are archetypal examples of endocrine disrupting compounds that may either affect the metabolism or action of hormones, or receptors involved in the etiology of obesity and with suspected links with metabolic diseases in humans [3, 21–24].

Using this model of exposure to a mixture of pollutants, we recently demonstrated that metabolic changes were highly dependent on sex in the absence of toxicity and weight change [25]. Notably, there was a marked deterioration of glucose tolerance in 12 week-old exposed mice females compared with age-matched non-exposed females, associated with the induction of hepatic expression of the estrogen sulfotransferase (EST/SULT1E1), a major estrogen-inactivating enzyme, and a reduced expression of the receptor alpha for estrogens (ERα) and of several estrogen-regulated genes, indicative of an impaired hepatic estrogen signalling in females exposed to low-dose food pollutants [25]. In the present study, we aimed at investigating the metabolic profile and liver functions in female mice fed HFSD at the earlier age of 7 weeks corresponding to the beginnings of obesity [25].

## Materials and Methods

### Diets and mice

All procedures were performed with the approval of the CECCAPP (C2EA-15; Comité d’Evaluation Commun au PBES, à AniCan, au laboratoire P4, à l’animalerie de transit de l’ENS, à l’animalerie de l’IGFL, au PRECI, à l’animalerie du Cours Albert Thomas, au CARRTEL INRA Thonon-les-Bains et à l'animalerie de transit de l'IBCP http://www.ifr128.prd.fr/pbes/site_web%20_ceccapp/Accueil_CECCAPP.html) which is the regional Committee of Ethics for Animal Experiments. Four week (wk) old female C57Bl/6J mice (Harlan, Le Marcoulet, France) were housed individually in polypropylene cages at 21°C with normal light/dark cycle and free access to water and standard chow. After one-wk acclimatization, females were randomly assigned to treatment groups, and they were fed a high fat-high sucrose diet (HFSD) containing or not a mixture of pollutants, as described in a recent study [25]. The mixture of pollutants was made of 2,3,7,8-tetrachlorodibenzo-*p*-dioxin (TCDD, CAS n° 1746-01-6; LGC-Promochem, Molsheim, France), Polychlorinated biphenyl (PCB) 153 (CAS n°35065-27-1), Bisphenol A (BPA, CAS n°80-05-7) and Di-[2-ethylhexyl]-phthalate (DEHP, CAS n°117-81-7) (all from Sigma-Aldrich, Saint-Quentin Fallavier, France). Each pollutant was used at a dose grossly corresponding to the TDI reference dose of either the pollutant itself (BPA, [26]; DEHP, [27]) or representative congeners of dioxins and dioxin-like PCBs with TCDD [28] and non-dioxin-like PCBs with PCB 153 [29] (S1 Table). The mixture was referred to as TDI∆ [25]. To ensure that animals received the correct amount of polluted food and were fed *ad libitum*, 1g of contaminated food /17 g body weight (bw) was distributed per day and completed by pollutant-free diet. To prepare contaminated food, pollutants dissolved in DMSO were diluted in corn oil to ensure good distribution in the food. Pollutant-free diet was prepared exactly as the pollutant-containing diet except that no pollutants were dissolved in DMSO. The compositions of the diets are indicated in the supporting information (S2 Table). The diet containing or not the mixture of pollutants was given to 5-wk old females for 5 wks before mating with adult standard chow-fed males and then along gestation and lactation. After weaning, F1 descendants were fed the same diet than their dams (S1 Fig). Mice fed HFSD without pollutant were designated as F0-HF0 for the dams and F1-HF0 for their offspring, and mice fed HFSD containing the mixture of pollutants were designated as F0-HFp for the dams and F1-HFp for their offspring. A control group corresponded to female mice and their offspring fed a classical standard chow diet that contained no corn oil or DMSO (referred as Standard).

### Metabolic tests and insulin injection

F1 mice were tested for glucose tolerance (GT Test) at 7 wks as described [30]. To test for tissue insulin sensitivity, mice were intra-peritoneally (IP) infused with insulin (Insulatard 2mU/g body weight) or saline after 6 h of food withdrawal. After 15 min, insulin sensitive tissues including the liver and the gastrocnemius muscle were rapidly dissected and snap frozen in liquid nitrogen.

### Blood and tissue collection

Seven- and 12-wk-old F1 mice were euthanized after 6 h of food withdrawal. Blood was collected and liver and adipose tissues (visceral: periovarian + parametrial, vAT and subcutaneous inguinal depots, scAT) were removed and snap frozen in liquid nitrogen. We measured blood glucose concentrations (glucometer OneTouchUltra, Lifescan, Issy-Les-Moulineaux, France) and plasma levels of insulin (ALPCO insulin ultrasensitive ELISA, Eurobio, Courtaboeuf, France), adiponectin (R&D Systems Europe Quantikine, Lille, France), free fatty acids (FFA) (Sunred Biological Technology, Clinisciences), triglycerides (TG) (BioMérieux, Marcy-l’Etoile, France), and 17β -estradiol (Interchim, Cayman EIA kit, Montluçon, France). Triglycerides (TG) were also measured in liver samples after lipid extraction by the method of Bligh and Dyer [31]. All relevant comparisons were made within the same assay.

### Quantitative RT-PCR (RT-qPCR)

Total RNA was extracted from the frozen liver and ATs samples. RNA was analysed by real-time PCR as described previously [32] in the presence of specific primer pairs (S3 Table) with data normalized relatively to Gusb (beta-glucuronidase, for liver) or Hprt (hypoxanthine ribosyl transferase, for AT) mRNA expression levels.

### Western-Blotting analyses

Ten micrograms of proteins prepared from mouse liver and gastrocnemius muscle were separated by SDS-10% polyacrylamide gel electrophoresis and transferred to a polyvinylidene difluoride (PVDF) membrane, as described [25]. Immunoblotting was performed using rabbit polyclonal antibodies directed against ERα (sc-542, Santa Cruz Biotechnology, CliniSciences, Nanterre, France), EST (sc-292049, Santa Cruz Biotechnology), phospho-AKT/PKB (Ser473) (#4060, Cell Signaling Technology Europe, Leiden, The Nederlands) and total AKT/PKB (#9272, Cell Signaling Technology Europe), or mouse monoclonal antibodies directed against α-tubulin (sc-5286, Santa Cruz Biotechnology). After incubation with either anti-rabbit or anti-mouse IgG Horseradish peroxidase conjugate (BioRad, Marnes-la-Coquette, France), blots were revealed using the Luminata *Classico* Western HRP substrate (Millipore, Molsheim, France). Detection was performed using the *ChemiDoc*XRS+ Imaging system (BioRad) and results were analysed with Image Lab software (BioRad). Data yielded for each protein were normalized relatively to α-tubulin.

### *In-vivo* Magnetic Resonance (MR) Imaging protocol

Experiments were conducted on anesthetized 7 wk-old females fed either standard chow diet or HFSD, containing or not the mixture of pollutants (n = 6 per group) using a 7T small animal Bruker system (Bruker, Ettlingen, Germany). The detailed procedure is described in the supporting information (S1 Materials and Methods).

### Statistics

All statistical analyses were performed using one-way ANOVA, followed by post hoc testing with Fisher’s protected least square difference test (PLSD). Results were expressed as mean ± SE and differences were considered significant at *p*<0.05 using the group not exposed to pollutants as reference. The number of samples is given for each figure.

## Results

### Metabolic impact of low dose pollutants in 7-wk old exposed females fed HFSD. Comparison with the 12-wk old exposed females

By week 7, females fed HFSD were 24% heavier than standard chow fed mice with higher glycaemia values (+33%, p = 0.001) and plasma insulin levels (+134%, p = 0.02) (Table 1). TG levels were unchanged (not shown) and plasma estradiol levels were below the detection threshold of the assay (<6.6 ng/ml; not shown) and lower than values (ranging from 24 to 31 pg/ml) measured in 12-wk old HFSD-fed females [25]. As for 12-wk old mice [25], pollutant exposure did not impact blood glucose or plasma levels of insulin (Table 1), adiponectin, FFA, triglyceride (not shown). Plasma estradiol levels remained below the detection threshold of the assay (<6.6 ng/ml; not shown). Body weight (Table 1) or food intake (not shown) were also not modified by pollutants in 7wk-old female mice, as previously shown for the 12-wk old exposed females fed HFSD [25].

**Table 1:** Body weight and biochemical parameters.

Parameters	7 wk-old mice					
	Standard		F1-HF0		F1-HFp	
	value	n	value	n	value	n
body weight (g)	15.3 ± 0.7 *	5 litters (7 mice)	19.8 ± 0.5	11 litters (22 mice)	18.8 ± 0.3	9 litters (21mice)
glycaemia (mmol/L)	6.7 ± 0.6 *	5 litters (7 mice)	8.9 ± 0.4	11 litters (22 mice)	10.0 ± 0.5	9 litters (21mice)
insulinemia (ng/ml)	0.29 ± 0.04 *	5 litters (7 mice)	0.68 ± 0.10	10 litters (21 mice)	0.76± 0.10	9 litters (18 mice)

Results are means ± SE of n litters as indicated; * p<0.05 *versus* F1-HF0

**Fig 1:**
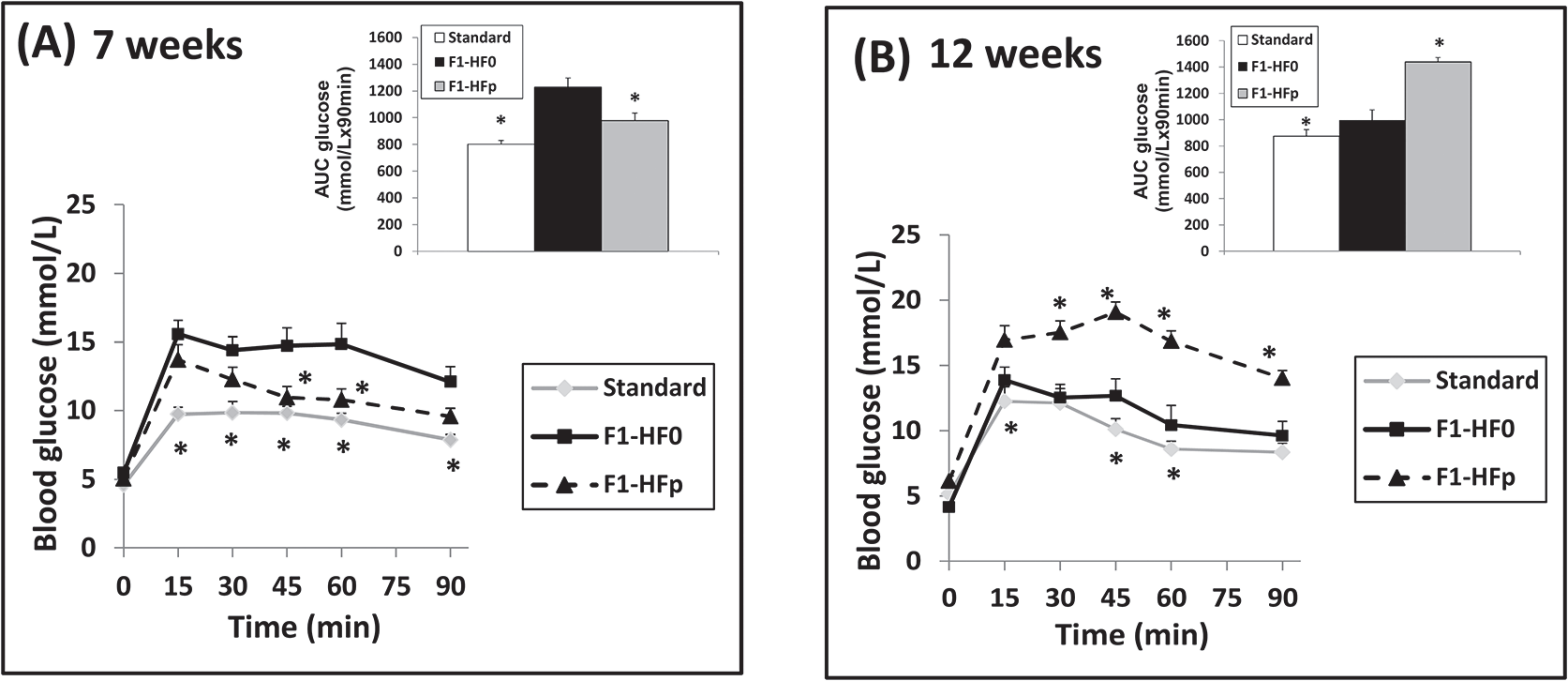
Glucose Tolerance Tests (GTTs) performed on 7 wk-old (A) and 12 wk-old (B) F1 female mice fed HFS diet containing (F1-HFp) or not (F1-HF0) the mixture of pollutants, or standard chow (Standard). Above each GTT curve, are shown the AUCs calculated from the GTT curves. Results are means ± SE of n = 5 (Standard), 7(HF0) and 9 (HFP) litters in (A) and n = 3 litters in B; *p<0.05 relatively to the F1-HF0 group.

Metabolic profiles were completed with GTTs. We observed that 7 wk-old exposed females had a better glucose tolerance with a significant decrease (-20%, p = 0.01) in the area under the curve (AUC) compared to age-matched females fed pollutant-free HFSD. Thus, pollutant-exposed mice fed HFSD ranged between mice fed standard chow and mice fed pollutant-free HFSD (Fig 1). These data are the compilation of the glycaemia values of 7 litters of HF0 females and 9 litters of HFp females from 4 independent experiments. A subset of mice (3 litters per group) were maintained until 12 wks of age and shown to have an aggravated glucose intolerance when exposed to pollutants compared to females fed pollutant-free HFSD (Fig 1), consistent with data previously reported and expressed per female mice [25]. Thus, these data suggested an age-dependent metabolic effect of the pollutants.

### Pollutant-exposed females had enhanced fatty acid oxidation in liver by week 7

**Fig 2:**
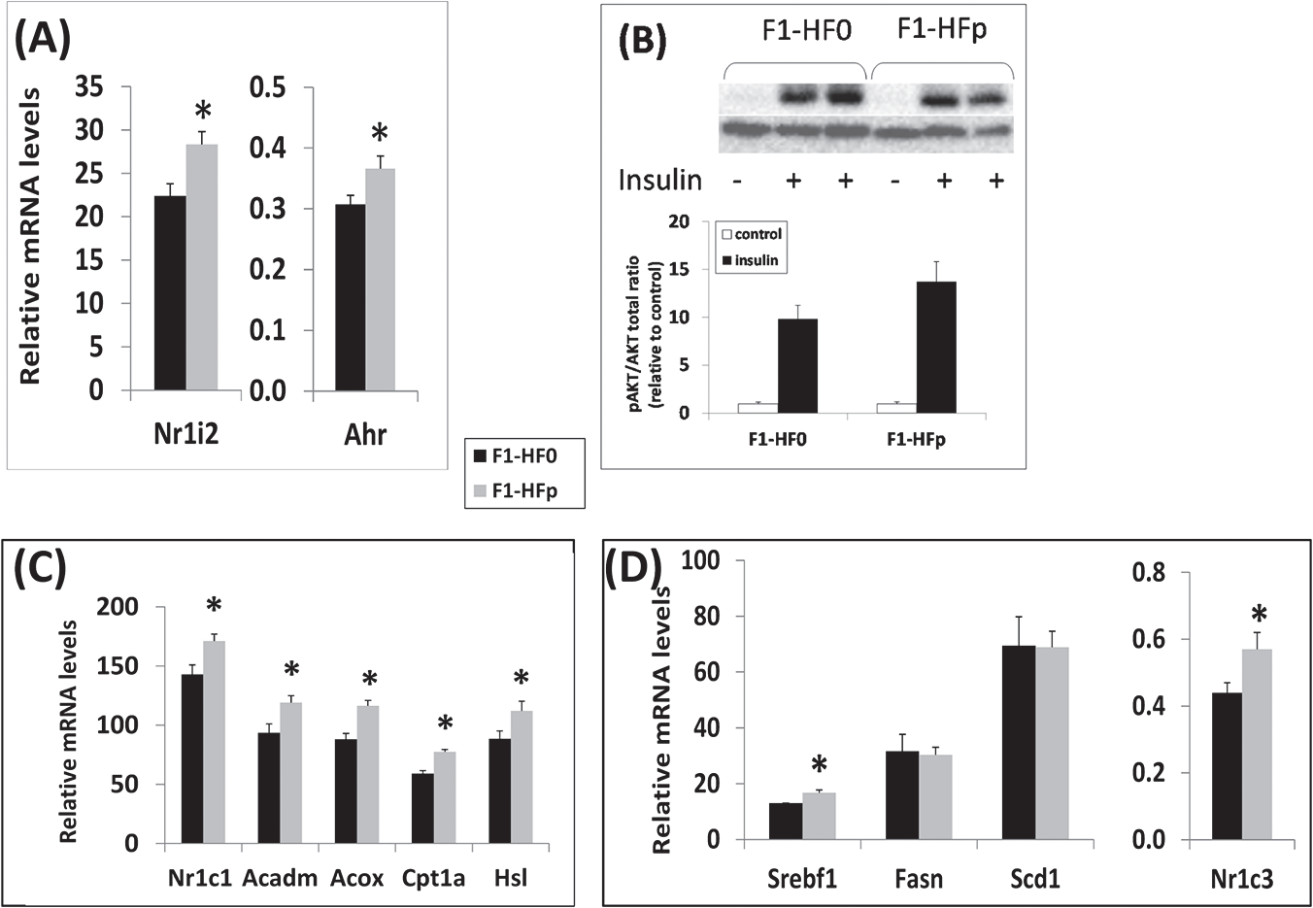
(A) Effect of the mixture of pollutants on hepatic mRNA expression of xenobiotic nuclear receptors; (B) Analysis by Western blot of the ratio between phosphorylated PKB/AKT versus total PKB/AKT in liver removed from F1-HF0 and F1-HFp mice stimulated or not by insulin. For quantification, each blot has been hybridized to α-tubulin and the graphs below represent means ± SE; n = 7–8 mice. (C) and (D) Hepatic expression of Nr1c1 and of several PPARα-regulated genes (C) and of genes encoding proteins involved in lipogenesis (D). Results are means ± SE; *p<0.05 relatively to the F1-HF0 group; n = 7 mice in (A), (C) and (D).

The liver being the major site of detoxification, it is a primary target upon pollutant exposure. At 7 wks of age, the expression of genes encoding the aryl hydrocarbon receptor, AhR and the pregnane X receptor, PXR (*Nr1i2*) (Fig 2A), known xenobiotic nuclear receptors were found significantly enhanced in the liver of exposed females fed HFSD, indicating patent response to pollutants. We then investigated in more details insulin action and glucose metabolism in the liver of these animals. Mice were infused with insulin and insulin action was monitored through measuring AKT/PKB serine phosphorylation levels as described [33, 34]. There was a 40% albeit not significant (p = 0.28) increase of AKT/PKB phosphorylation in the liver of the pollutant-exposed females (Fig 2B). In addition, we observed a significant higher expression levels of *Nr1c1* encoding peroxisome proliferator-activated receptor α (PPARα; + 20%; p = 0.02). There was as well an increased expression of several PPARα-regulated genes (Fig 2C) coding key proteins and enzymes involved in mitochondrial fatty acid import (Carnitine palmitoyltransferase 1α, *Cpt1a*; +31%, p<0.002), oxidation (medium-chain-acyl-Coenzyme A dehydrogenase, *Acadm*; +27%, p<0.02), and peroxisomal beta-oxidation (AcylCoA oxidase, *Acox*; +32%; p = 0.001). The expression of the gene encoding the hormone sensitive-lipase (HSL, + 27%; p = 0.04; Fig 2C), *Nr1c3* (encoding PPARγ; + 29%, p<0.05) and *SrebF1* (encoding SREBP1c; + 29%, p<0.05) were also significantly increased without any change in the levels of mRNA encoding stearoyl-CoA-desaturase-1 (*Scd1*) and fatty acid synthase (*Fas*) (Fig 2D). These results suggested some modifications in lipid metabolism, with increased fatty acid oxidation but without major change in lipogenesis. In agreement, hepatic triglycerides levels were not modified by pollutant exposure (not shown). We also found that the mRNA expression of genes encoding Glucose-6-phosphatase (*G6pc*) and phosphoenolpyruvate carboxykinase (*Pepck*) was unaffected (not shown), suggesting that gluconeogenesis was probably not impacted.

### Hepatic Sult1e1 expression is not altered in 7-wk old exposed females

**Fig 3:**
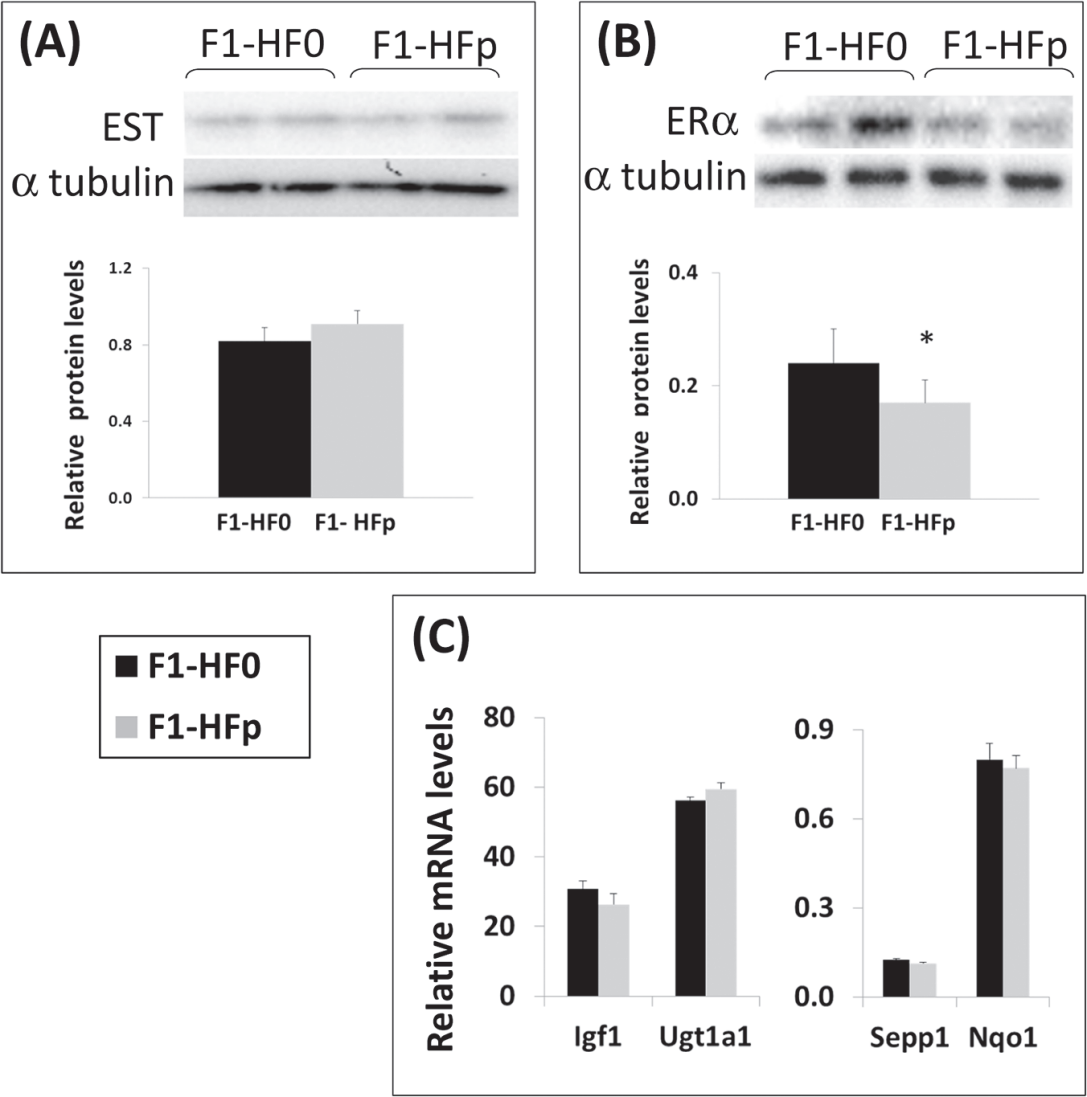
EST/ Sult1e1 (A) and ERα (B) were studied by Western blotting analyses. The graphs below correspond to the quantification and represent means ± SE; *p<0.05 relatively to the F1-HF0; n = 7–8 mice. (C) mRNA expression of some estrogen-regulated genes in 7wk-old female mice fed HFS diet containing (F1-HFp) or not (F1-HF0) the mixture of pollutants. Results are means ± SE (n = 7 mice).

We next determined if estrogen signalling was impaired in the liver of pollutant-exposed females by week 7 as previously observed at 12 wks of age [25]. However and contrasting with 12–wk old mice fed HFSD exposed to the mixture of pollutants, we did not detect any change in the protein levels of EST/SULT1E1 (Fig 3A). In addition, although a significant (p = 0.02) decrease in estrogen receptor (ERα) protein content was observed (Fig 3B), the mRNA levels of the estrogen-regulated genes *Igf1*, *Sepp1*, *Nqo1* and *Ugt1a1*, which expressions decreased in the liver of 12-wk old exposed females fed HFSD [25], were not affected by pollutant exposure at 7 wks of age (Fig 3C). These results suggested therefore different effects of pollutant exposure on hepatic estrogen signalling which may depend on the duration of the exposure to the pollutant-containing HFSD and /or on the age at which the female mice are studied.

### Pollutant exposed females exhibited enhanced lean mass and reduced fat mass by week 7

**Fig 4:**
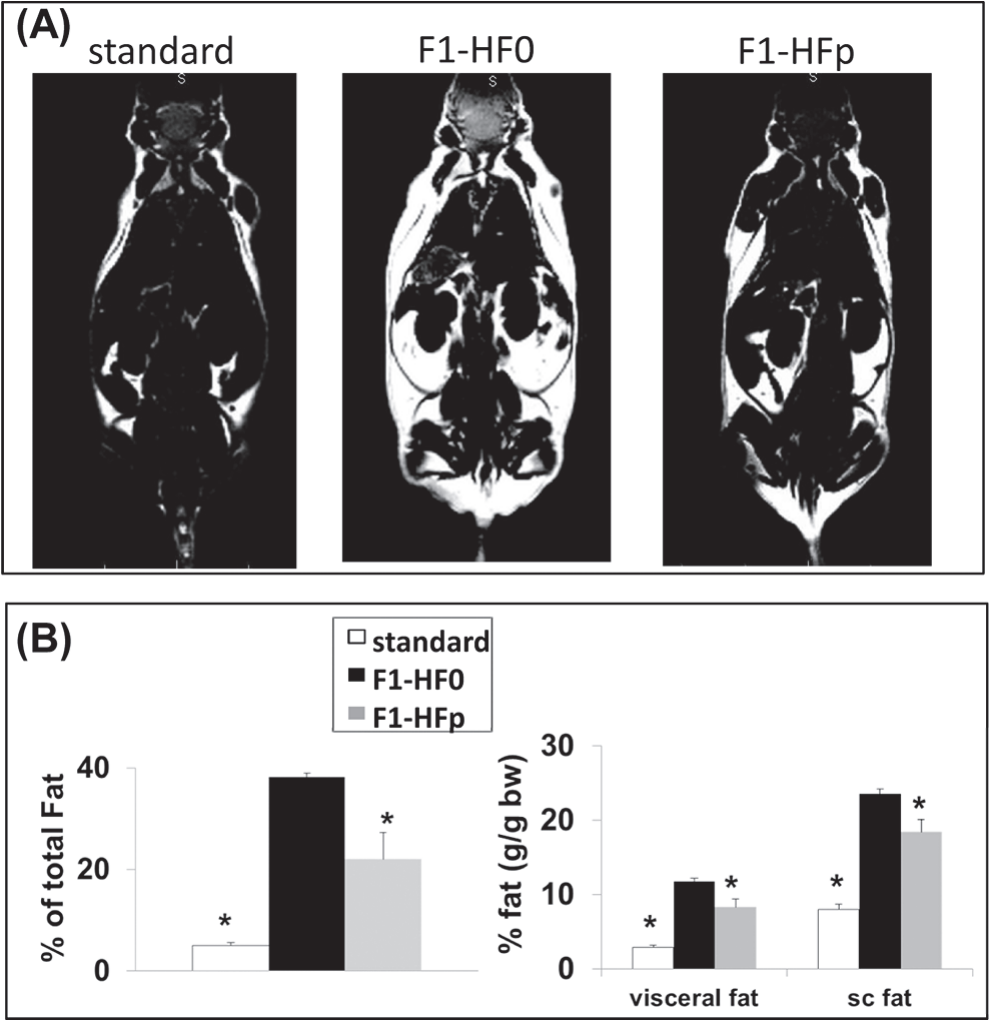
MRI figures and graphs. (A) Representative coronal fat image of females fed standard chow and from F1-HF0 and F1-HFp groups, showing the relatively less important fat mass in the F1-HFp group, especially in the subcutaneous localization. These differences were confirmed by the whole-body spectrum analysis, and by the subcutaneous (sc) and visceral fat mass quantification in both groups (B). Results are means ± SE (n = 6 mice/group). *p<0.05 relatively to the F1-HF0 group.

**Fig 5:**
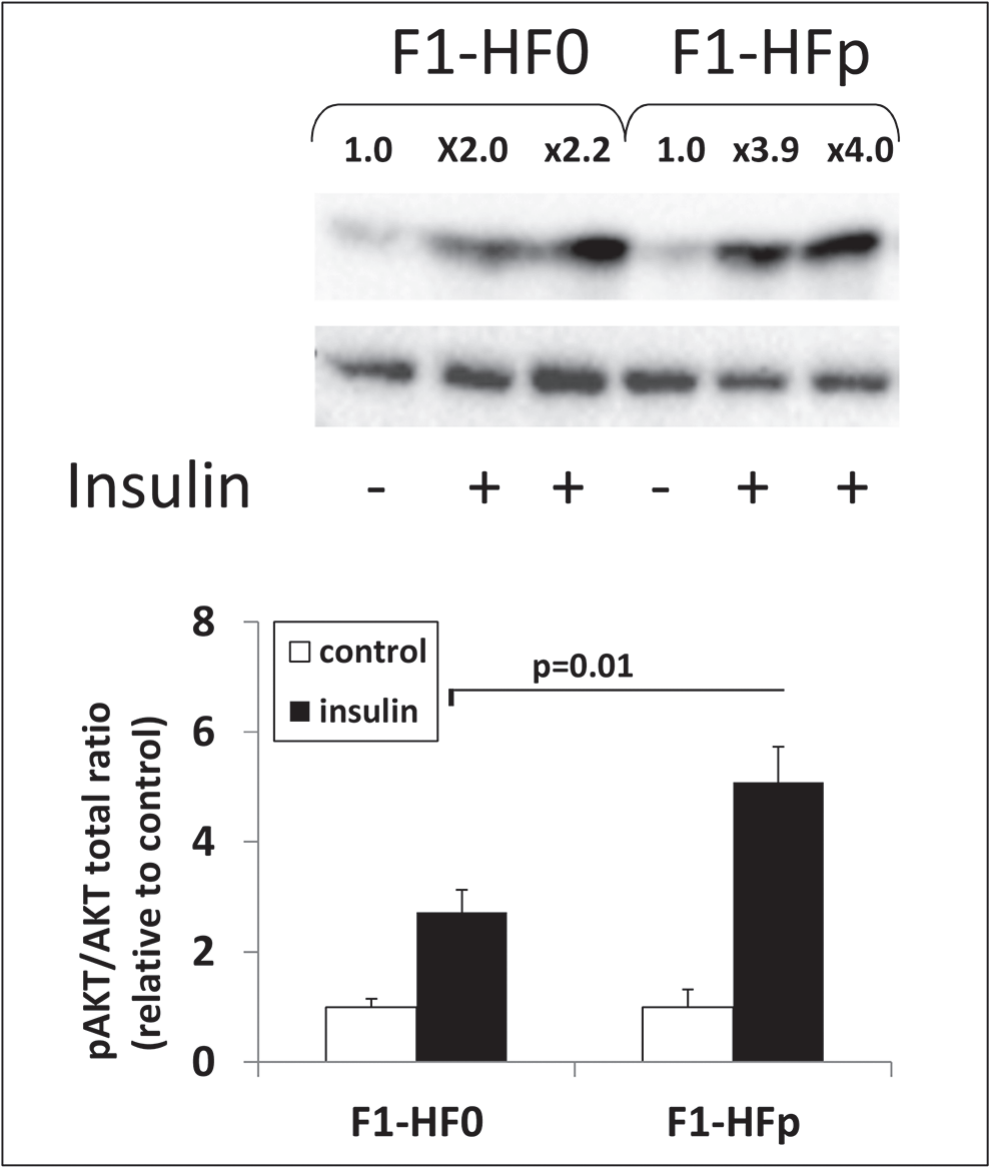
Western blot analysis of the ratio between phosphorylated PKB/AKT versus total PKB/AKT in the gastrocnemius muscle removed from F1-HF0 and F1-HFp mice stimulated or not by insulin (the fold stimulation by insulin, relative to the mean of the corresponding control without insulin was assigned above each band shown). For quantification, each blot has been hybridized to α-tubulin and the graphs below represent means ± SE; *p<0.05 relatively to the F1-HF0 group; n = 7–8 mice.

To get further insight into the metabolic characterization of the 7-wk old exposed female mice fed HFSD, we carried out whole-body magnetic resonance imaging (MRI) analysis. Interestingly, we observed a significant increase (+ 20%; p = 0.02) of lean mass in these pollutant-exposed obese females, while both visceral and subcutaneous fat tissues were significantly decreased (Fig 4). This effect was not observed in age- and treatment-matched male mice (S2 Fig) nor was glucose tolerance affected by pollutant exposure in these males (not shown). Because the skeletal muscle is the primary tissue for glucose disposal [35], insulin sensitivity was monitored by measuring AKT/PKB serine phosphorylation in muscles sampled 15 min after insulin infusion, as described for the liver. We found a significant enhancement of AKT/PKB phosphorylation (+ 80%; p = 0.02) in the muscles recovered from pollutant-exposed mice as compared to age-matched non-exposed females (Fig 5), indicating a clear improvement of insulin sensitivity in the muscle of exposed animals.

### Expression levels of inflammatory markers in the adipose tissues

**Fig 6:**
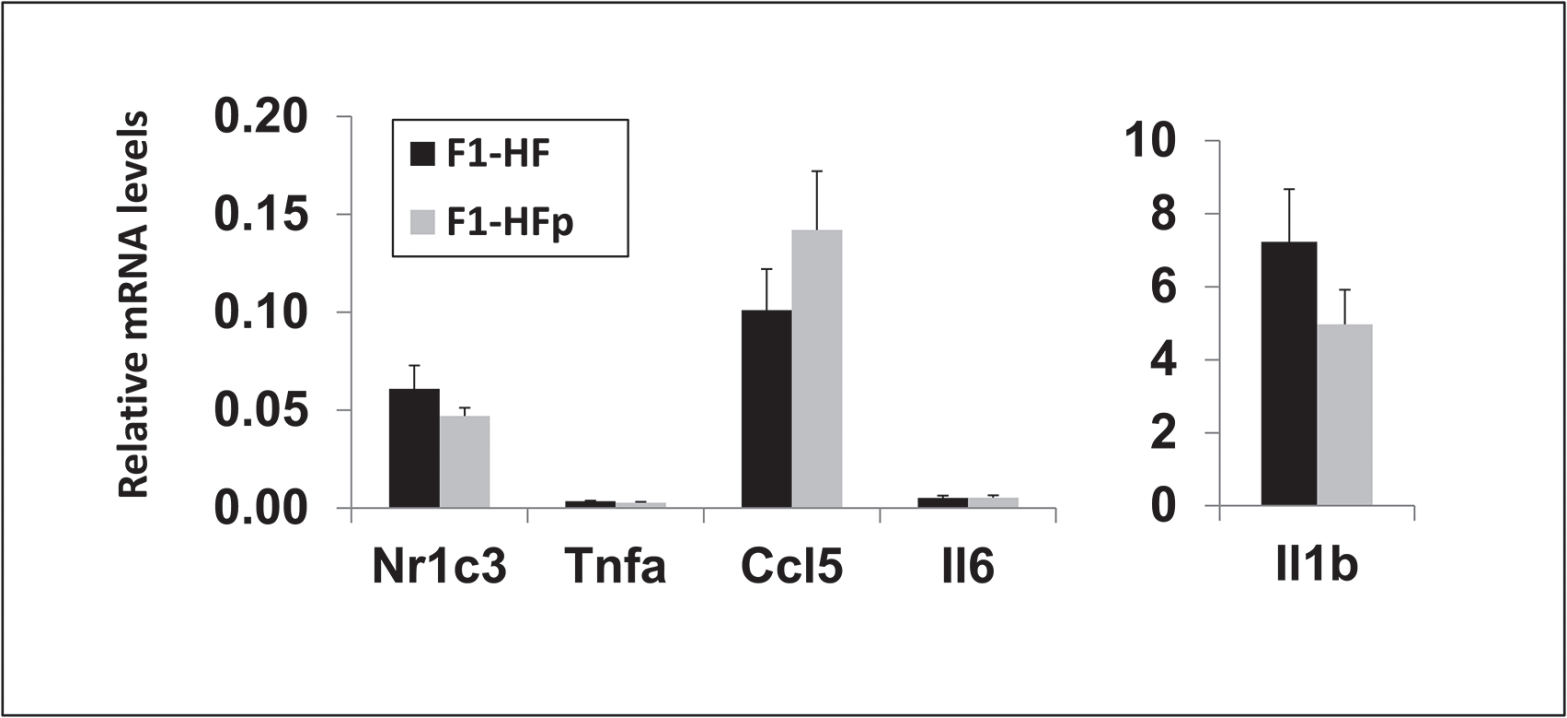
mRNA expression of Nr1c3 and genes encoding inflammatory markers in visceral adipose tissues in 7 wk-old female mice fed HFSD. Results are means ± SE; *p<0.05 relatively to the F1-HF0 group; n = 9–10 mice for Nr1c3, Tnfα and Il6; n = 5 mice for Ccl5 and Il1b.

**Fig 7:**
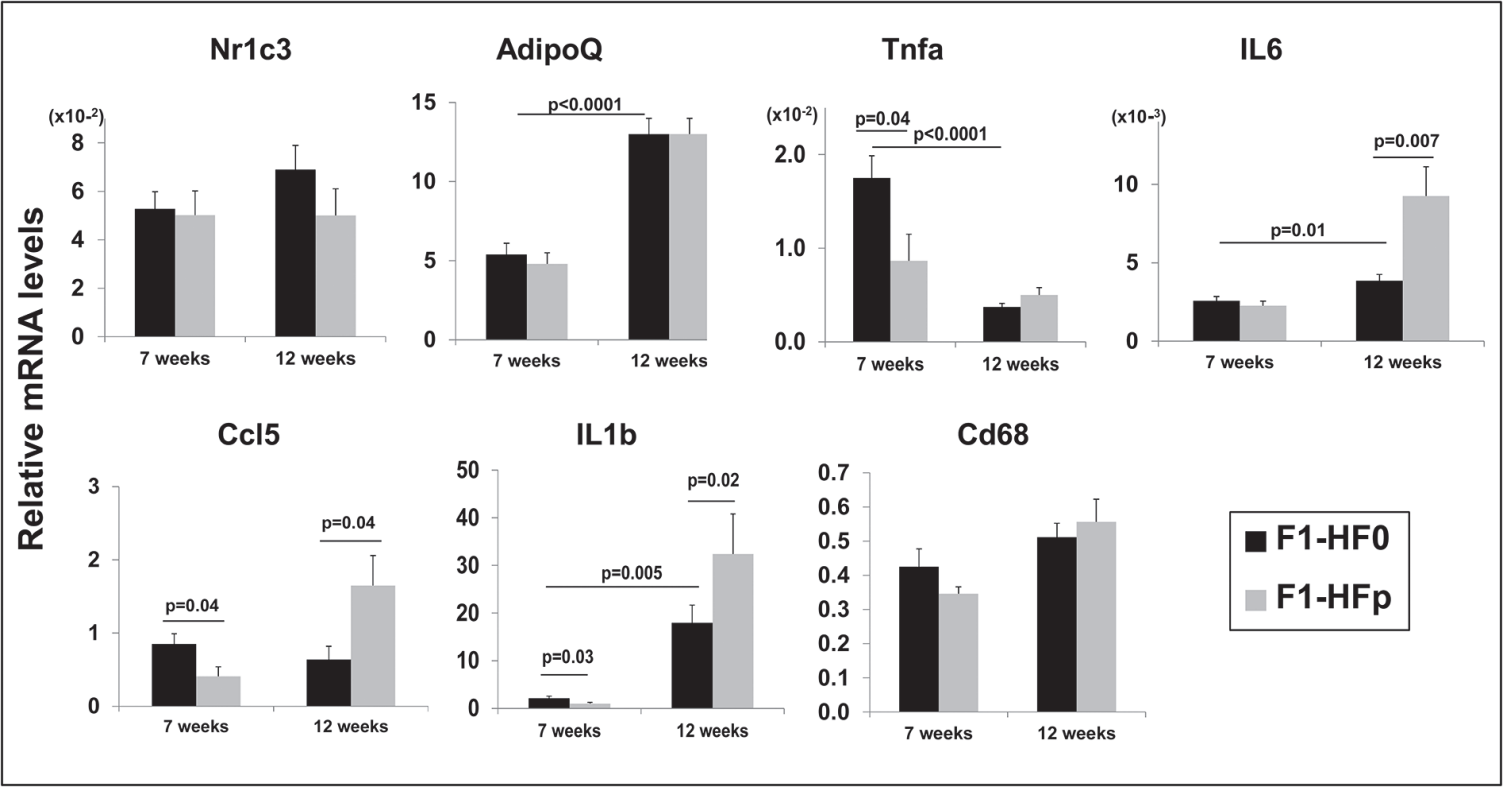
mRNA expression of Nr1c3, AdipoQ, CD68 and genes encoding inflammatory markers in the subcutaneous adipose tissues of 7 and 12 wk-old female mice fed HFSD. Results are means ± SE; *p* values are reported in the figure for significance; n = 8–12 mice for Nr1c3, Tnfα, CD68, AdipoQ and Il6 and n = 5 mice for Ccl5 and Il1b in the samples from 7-wk old mice; n = 5–6 mice for Nr1c3 and n = 9–14 mice for the other genes in the samples from 12-wk old mice.

Since pollutants have been described to induce the inflammatory pathway in various tissues [36, 37] including the adipose cells [38], and considering the importance of the inflammatory phenotype in metabolic diseases [39], we monitored the expression levels of genes encoding various inflammatory markers in visceral (vAT; Fig 6) and subcutaneous (scAT; Fig 7) adipose tissues. We first found that the expression level of *Nr1c3*, encoding PPARγ, was not modified (Figs 6, and 7) indicating that exposure to pollutants did not probably have a major impact on adipose differentiation. The expression of genes encoding tumor necrosis factor α (*Tnfa*), CCL5/Rantes (*Ccl5*) and interleukin 1β (*Il1b*) all known inflammatory markers, while significantly decreased in the scAT (Fig 7) was not impacted in the vAT of 7-wk old F1-HFp females (Fig 6). These data suggested a reduced inflammation at least in the scAT consistent with an improvement of glucose intolerance in 7-wk old female mice exposed to pollutants and fed a HFS diet.

To get further evidences of a link between the metabolic phenotypes observed at 7 and 12 wks and the inflammatory state of the adipose tissues, we monitored the expression levels of genes encoding CCL5, IL1β, and TNFα in the scAT and vAT of the 12-week old F1-HF0 and F1-HFp groups. Interleukin 6 (*Il6*) was also surveyed at both ages. None of the cytokines had their expression level impacted by pollutant exposure in vAT at both ages surveyed (Fig 6 and not shown). Interestingly, we observed a significant enhancement in the expression levels of genes encoding CCL5, IL6 and IL1β in the scAT of the 12-wk old F1-HFp females, while *Il6* expression was not altered by wk 7 (Fig 7). In addition, genes encoding inflammatory cytokines IL6, IL1β and CCL5 (in the F1-HFp mice) showed expression levels significantly higher in scATs at 12- than at 7-wks (Fig 7). On the contrary, expression levels of the gene encoding TNFα decreased strongly between the ages of 7 and 12 together with the lack of impact of the pollutant exposure by wk 12 (Fig 7). Finally, we monitored the expression levels of the gene encoding CD68, a marker of macrophage infiltration in ATs [40]. However, we found no impact in pollutant-exposed females versus their time-matched unexposed females (Fig 7). Of note, the expression levels of *AdipoQ* (encoding adiponectin) in scAT were more than doubled between the ages of 7 and 12 although not impacted by pollutant exposure (Fig 7) again suggesting that adipose differentiation was not targeted by the pollutant mixture in scAT.

## Discussion

The present study illustrates the complexity of the impact of a life course exposure to low-dose pollutants on metabolic disorders induced by HFSD in female mice. The model of exposure recently developed in our laboratory [25] is original because of life-long exposure, encompassing maternal life (preconception, gestation and lactation). In addition, animals are exposed to a mixture of classical environmental pollutants in the range of the tolerable daily intake (TDI), thus without expected adverse health impact when each pollutant is considered individually. Pollutants of the mixture comprised endocrine disruptors with different modes of actions, and of high concern for human health because of their links with metabolic diseases in epidemiological and experimental studies [20–22, 24].

With HFSD, the onset of obesity was gradual in our experimental model and was set up by week 12 in females with a 40% increase in body weight, enhanced levels of fasting glycaemia and plasma insulin levels [25]. While exposure to pollutants did not impact these parameters, we have observed significant sex-dependent metabolic changes by week 12, in absence of toxicity and weight modifications. Specifically, there was a marked deterioration of HFSD-induced glucose intolerance in females that was not observed in males [25]. Within the present study, we aimed at further characterizing exposed HFSD-fed female mice by week 7 when first signs of obesity could be observed. Surprisingly, the glucose intolerance induced by the HFS diet was improved in pollutant exposed females, and a reduced fat mass and increased lean body mass (without any weight change) was observed. It indicated that the aggravation of glucose intolerance, observed in 12-wk old pollutant-exposed obese mice, was preceded by a period of improvement of glucose tolerance. Consistent with the alleviation of glucose tolerance by week 7, we observed enhanced insulin sensitivity in the gastrocnemius muscle and a reduced expression of inflammatory markers in subcutaneous adipose tissue that was not observed at 12 weeks of age (i.e., there was even a significant increased expression of several inflammatory markers at this age). In addition, gene expression analysis in the liver suggested higher oxidation of fatty acids but no alteration of gluconeogenesis and no effect on steatosis. These data occurred with no concomitant alteration of hepatic estrogen metabolism and no change in the expression of the estrogen-regulated genes studied despite a reduction of the hepatic ERα protein content. These data also suggested that the observed metabolic improvement was more likely due to effects on muscle and/or adipose tissue, rather than on the liver. Moreover, they pointed to a possible key role of the adipose tissues in the kinetics of the described events especially if considering the enhanced expression of inflammatory markers in the subcutaneous adipose tissue by week 12 (this study) together with the concomitant and previously reported aggravation of glucose intolerance [25].

Collectively, our data fit the hypothesis of a biphasic metabolic impact of pollutants: a first apparent positive impact that is followed by a negative impact on glucose intolerance upon aging. One explanation could well be linked to body fatness taking into consideration that the adipose tissue acts as a reservoir for lipophilic pollutants and consequently may modulate their pharmacokinetics and toxicity [10]. Indeed, it was demonstrated that mouse size is achieved around 5–6 weeks of age [41], which means that the 29% extra-weight taken up between the 7 and 12 week-old HFSD fed females [25] grossly corresponds to fat mass, mostly in the white adipose tissues. Increases in the adipose tissue mass will result in an enhanced storage of the lipophilic TCDD and PCB153 thereby prolonging their half-life length as described [42, 43]. Therefore, as mice get older their POPs body burden will enhance. However, based on the measurement of adipocyte differentiation markers, it does not seem that adipocyte differentiation is targeted by pollutants in our experimental model. These data are consistent with the lack of gain weight under pollutant exposure contrasting with obesogen compounds which elicit enhanced expression of PPARγ and increased weight gain in the exposed animals [44]. Interestingly, we evidenced significant changes in the inflammatory state of the subcutaneous adipose tissue in response to the exposure to pollutants with a decrease by week 7 and an increase by week 12, in the absence of any variations in *Cd68* expression levels suggesting no massive macrophage infiltration. These data further strengthen the hypothesis that inflammation is one way by which pollutants can disrupt adipose tissue function and contribute to metabolic diseases [38, 39]. Further studies will help to understand if the kinetics of the induction of inflammation in the subcutaneous adipose tissue plays a role in triggering the kinetics of changes of glucose intolerance (i.e., alleviation at 7 weeks and aggravation at 12 weeks), and its link with the hepatic insulin resistance in the 12 week-old HFSD females lifelong exposed to pollutants [25]. Nonetheless, this study highlights that dysfunction of the subcutaneous adipose tissue may also (and not only the visceral adipose tissue) possibly trigger metabolic abnormalities [45]. Whether it is related to a differential distribution of the pollutants within the fat pads remain to be explored. Collectively, our data are consistent with the conclusions of experimental studies with BPA in rats [18] and PCB153 in mice [19] suggesting that intake of a high-fat diet could be a trigger initiating adverse metabolic effects. Certainly, measurement of pollutants and their metabolites within the fat pads of the exposed females as well as feeding mice with a standard chow diet containing or not the mixture of pollutants will help to better comprehend this intriguing biphasic metabolic impact of pollutants. It may as well be of interest to study intestinal permeability whom integrity has been shown to be altered by BPA [46] and PCBs including PCB153 [47], and intestinal microflora because these parameters strongly influence lipid metabolism and metabolic health [48, 49].

A not mutually exclusive explanation for this apparent biphasic effect of pollutants may rely on plasma E2 levels. Indeed, E2 levels varied from 24 to 31 pg/ml in 12-week old females fed HFSD [25], which is consistent with values reported in the literature for standard C57BL/6 strain mice [50, 51] while they were below detectable levels at 7 weeks of age in females fed HFSD. It was indicative of differences in the hormonal environment between obese mice at 7 and 12 weeks of age in our experimental conditions. Besides, it is known that estrogens protect against diet-induced hepatic insulin resistance and glucose intolerance [33, 52, 53]. Hence, models of estrogen deficiency in both humans and rodents well illustrated the importance of estrogens in regulating energy homeostasis and insulin sensitivity when estrogens concentrations stay within a tight physiological range [54]. Thus, one could hypothesize that the estrogeno-mimetic effect of some of the pollutants (or their metabolites) might occur in a context of low estradiol levels, protecting the 7-week old animals against the deleterious metabolic effects of HFSD. In contrast, at 12 weeks of age, when circulating levels of estrogens are higher, pollutants may contribute to the induction of EST/SULT1E1 reducing estrogen signalling. Under such conditions, liver would no more benefit from the protective action of estrogens and, consequently, insulin resistance would be aggravated.

While additional works are thus needed to validate this hypothesis, it is important to note that interplay between metabolism, pollutants and estrogen action is already well documented. There have been several reports indicating that the adverse effects of endocrine disruptors depend on the estrogenic context. Actually, a large number of genes involved in energy metabolism present ERα-binding sites in their promoters [55] and the pleiotropic actions of estrogens are mediated through interactions with many signalling pathways [56]. Consistently, it was shown that PPARα functions on obesity could be enhanced in estrogen-deficient states e.g. ovariectomized females [57]. Another example is given with the dioxins. Indeed, there is an extensive crosstalk between the ER and the aryl hydrocarbon receptor AhR, resulting in dioxins altering estrogen regulated genes and modulation of AhR activity by ERα [58, 59]. Noteworthy, the expression of the genes encoding AhR and PPARα was enhanced in the liver of 7-week pollutant-exposed females while such modification was only observed in the liver of 12 week-old males (not females) exposed to the same mixture of pollutants [25]. But, contrary to what was observed in 7-week pollutant-exposed females, genes encoding proteins related to fatty acid oxidation had their expression levels unchanged in 12 week-old exposed males, again supporting the hypothesis of a dialog, involving the estrogens, with the pathways activated by pollutants.

The pollutants present in the mixture have not been tested individually in our experimental model, making it difficult to conclude whether the effects of the mixture are additive, synergic or antagonist. We think however that the enhanced expression of genes encoding AhR and PPARs may result from exposure to dioxin and phthalate, respectively, on the basis of the reported actions of these compounds [2, 12, 24]. It may thus indicate that high estrogen levels such as those present in 12 week-old adult female mice interfere with the ability of pollutants present in the mixture to induce higher expression levels of genes encoding AhR and PPARα. Further studies are warranted to challenge this hypothesis. In addition, interactions of dioxin and phthalate with the other pollutants present in the mixture may be considered as well, specifically regarding BPA which has been shown to activate the estrogenic pathway but also the PPAR pathway [12, 24]. In conclusion, we here confirmed and extended our previous findings [25] demonstrating that exposure to a mixture of food pollutants at doses around the Tolerable Daily Intake range does indeed have metabolic consequences in a rodent model, and that effects are not only sex-dependent but also age-dependent.

## Supporting Information

S1 Fig Experimental protocol developed in the present study. (TIF)

S2 Fig MRI graphs for male mice. Whole-body spectrum analysis, and subcutaneous (sc) and visceral fat mass quantification in 7-wk old standard chow fed mice and mice fed HFSD without (F1-HF0) or with the mixture of pollutants (F1-HFp). Results are means ± SE (n = 6 mice/group). *p<0.05 relatively to the F1-HF0 group.
(TIF)

S1 Materials and Methods MRI procedure. (DOCX)

S1 Table Reference doses of the pollutants present in the mixture and doses added to the high fat high sucrose diet. (DOCX)

S2 Table Composition of the diets used in the present study (modified from TD.99249, Harlan). (DOCX)

S3 Table List of primers used for quantitative PCR. (DOCX)
