# Supplementary figures and images for: Metabolic Outcome of Female Mice Exposed to a Mixture of Low-Dose Pollutants in a Diet-Induced Obesity Model
Source: PLoS One. 2015 Apr 24;10(4):e0124015. doi: 10.1371/journal.pone.0124015 (PMC4409066; doi:10.1371/journal.pone.0124015)

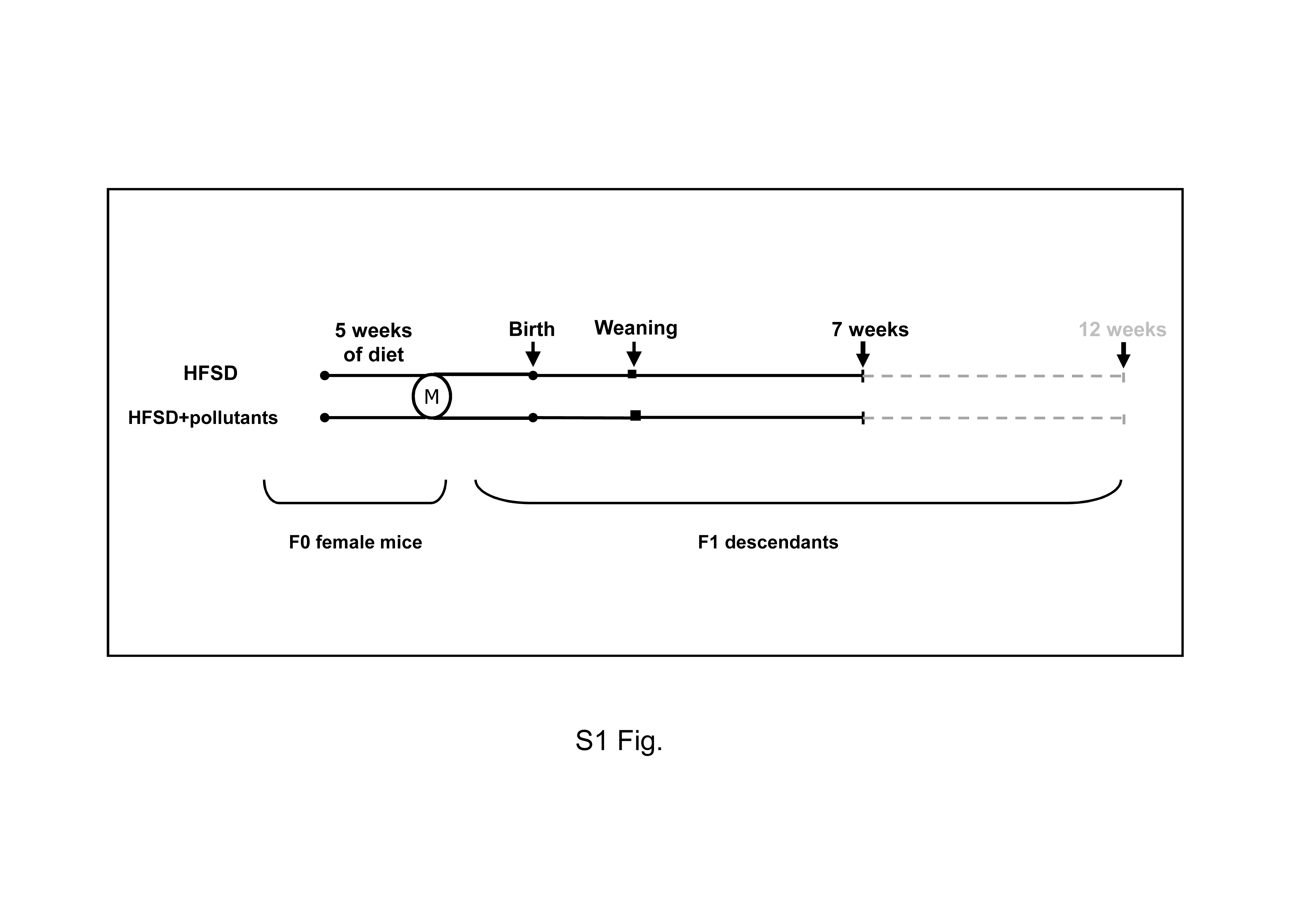

Supplement: S1 Fig — (TIF) [file pone.0124015.s001.tif]

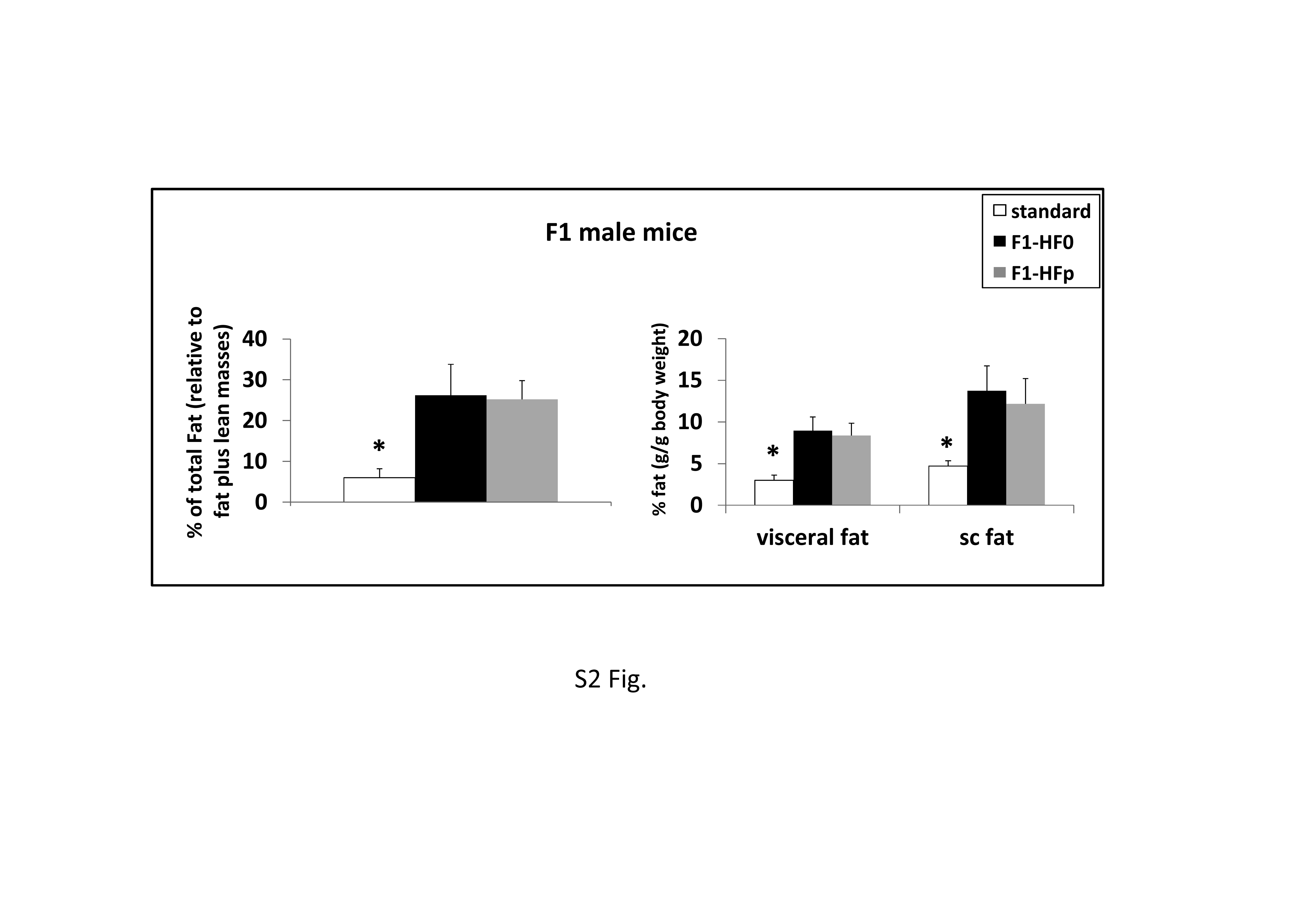

Supplement: S2 Fig — Whole-body spectrum analysis, and subcutaneous (sc) and visceral fat mass quantification in 7-wk old standard chow fed mice and mice fed HFSD without (F1-HF0) or with the mixture of pollutants (F1-HFp). Results are means ± SE (n = 6 mice/group). *p<0.05 relatively to the F1-HF0 group. (TIF) [file pone.0124015.s002.tif]
