# Supplementary material for: Metabolic Outcome of Female Mice Exposed to a Mixture of Low-Dose Pollutants in a Diet-Induced Obesity Model
Source: PLoS One. 2015 Apr 24;10(4):e0124015. doi: 10.1371/journal.pone.0124015 (PMC4409066; doi:10.1371/journal.pone.0124015)
Supplement: S1 Materials and Methods — (DOCX) [file pone.0124015.s003.docx]

**S1 Materials and Methods**: MRI procedure

The procedure is derived from Ranefall and collaborators (Ranefall et al., 2009). Anesthesia was induced by inhalation of a mixture of air and 3% isoflurane and maintained by a mixture of air containing 1.5% isoflurane. Body temperature was maintained using a circulating water circuit and respiration was continuously monitored over the entire protocol. Experiments were performed on a 7T small animal Bruker system (Bruker, Ettlingen, Germany) equipped with a 12-cm actively shielded bore and 400 mT/m gradient set. A pressure sensor was used for the respiratory signal and respiratory triggering was achieved with the Rapid Biomed system (RapidBiomedical, GmbH, Rimpar, Germany). MR acquisitions were performed with the whole-body emission-reception mouse coil (72 mm). First, a reference scan (3 directions) was acquired to adjust shim and frequency parameters over the entire body and to position the coronal stack for whole body fat analysis (subcutanous and visceral fat, respectively). For the water image, a 2D T1-weighted respiratory gated spin echo sequence (MSME) with fat suppression was acquired with the following parameters: TR/TE= 700/8 .1 msec, a matrix size of 256*128 for a voxel size of 350x350x750 µm^3^. Fat images were acquired using the cloned MSME sequence of the water image with a B1 shift frequency of 1050 Hz (the water-fat frequency difference at 7T). A whole body spectrum (SinglePulse, with the following parameters TR=1000 ms, NA 120, TA 2 min) was then acquired to determine fat and water ratio. Spectrum analysis was done with Topspin (Bruker). After phase and baseline correction, areas of the water and fat peaks were measured and expressed as a % of sum of peak integrals. Image analysis of intra-abdominal and subcutaneous fat volumes were performed with Amira® (FEI Visualization Sciences Group, Merignac, France) using a semi-automatic segmentation tool and standard anatomic landmarks (Ranefall et al, 2009) after exclusion of the head, the inferior legs and the tail. Weights are derived using a fat density of 0.9255.

RANEFALL P, BIDAR AW, HOCKINGS PD. 2009. [Automatic segmentation of intra-abdominal and subcutaneous adipose tissue in 3D whole mouse MRI.](http://www.ncbi.nlm.nih.gov/pubmed/19711401) J Magn Reson Imaging. Sep;30(3):554-60.
