# Supplementary material for: Metabolic Outcome of Female Mice Exposed to a Mixture of Low-Dose Pollutants in a Diet-Induced Obesity Model
Source: PLoS One. 2015 Apr 24;10(4):e0124015. doi: 10.1371/journal.pone.0124015 (PMC4409066; doi:10.1371/journal.pone.0124015)
Supplement: S1 Table — (DOCX) [file pone.0124015.s004.docx]

**S1 Table:** Reference doses of the pollutants present in the mixture and doses added to the high fat high sucrose diet

| **values/kg bw/d** | TDI | TDI Δ |
| --- | --- | --- |
| TCDD | 1-4 pg | 2 pg |
| PCB153 | 20 ng | 80 ng |
| BPA | 50 µg | 5 µg |
| DEHP | 50 µg | 50 µg |
