# Supplementary material for: Metabolic Outcome of Female Mice Exposed to a Mixture of Low-Dose Pollutants in a Diet-Induced Obesity Model
Source: PLoS One. 2015 Apr 24;10(4):e0124015. doi: 10.1371/journal.pone.0124015 (PMC4409066; doi:10.1371/journal.pone.0124015)
Supplement: S2 Table — (DOCX) [file pone.0124015.s005.docx]

**S2 Table: Composition of the diets used in the present study (modified from TD.99249, Harlan).**

|  | **High Fat diet (TD.99249, Harlan)** | | **HFSD** | |
| --- | --- | --- | --- | --- |
|  | % mass | %kcal | % mass | %kcal |
| diet fat | 36.1 |  | 34.3 |  |
| added corn oil |  |  | 5.1 |  |
| **Total fat** | 36.1 | 58.7 | 39.4 | 63.0 |
| **Carbohydrate** | 35 (maltodextrin 50% + sucrose 50% | 25.5 | 33.2 (maltodextrin 50% + sucrose 50% | 23.6 |
| **Protein** | 19.8 | 15.7 | 18.8 | 13.4 |
| **kcal/g** | 5.4 | | 5.6 | |
