# Supplementary material for: Metabolic Outcome of Female Mice Exposed to a Mixture of Low-Dose Pollutants in a Diet-Induced Obesity Model
Source: PLoS One. 2015 Apr 24;10(4):e0124015. doi: 10.1371/journal.pone.0124015 (PMC4409066; doi:10.1371/journal.pone.0124015)
Supplement: S3 Table — (DOCX) [file pone.0124015.s006.docx]

**S3 Table**: List of primers used for quantitative PCR

|  |  | **sequences: 5'->3'** | |
| --- | --- | --- | --- |
| **gene** | **RefSeq Accession Number** | **sense** | **antisense** |
| ***Acadm*** | NM_007382 | **CAT-TTG-GAA-AGC-TGC-TAG-TG** | **CCA-TAG-CCT-CCG-AAA-ATC-TG** |
| ***Acox1*** | NM_015729,NM_001271898 | **TGA-GGG-CGC-CAG-TCT-GAA-ATC** | **TGA-AGC-TCA-GGC-AGC-TCA-CTC** |
| ***AdipoQ*** | NM_004797; NM_001177800 | **AGG-CCG-TGA-TGG-CAG-AGA-TG** | **CTT-CTC-CAG-GTT-CTC-CTT-TCC-TGC** |
| ***Ahr*** | NM_013464.4, NM_013149.2 | **TCA-TCT-GGT-TTC -CTG-GCA-ATG-AAT** | **ATA-AGC-TGC-CCT-TTG-GCA-TC** |
| ***Ccl5*** | NM_031116.3 | **CTT-GCA-GTC-GTC-TTT-GTC-AC** | **GAC-TAG-AGC-AAG-CAA-TGA-CAG** |
| ***Cd68*** | NM_009853 | **AGC-ATA-GTT-CTT-TCT-CCA-GC** | **ATG-ATG-AGA-GGC-AGC-AAG-AG** |
| ***Cpt1a*** | NM_013495 | **TCT-GAG-CCA-TGG-AGG-TTG-TC** | **CATCATGGCTTGTCTCAAGTGC** |
| ***Esr1*** | NM_000125;NM_001122740;NM_001122741;NM_001122742 | **TGT-TTG-CTC-CTA-ACT-TGC-TC** | **CCT-TCT-CTT-CCA-GAG-ACT-TC** |
| ***Fasn*** | NM_07988 | **GTG-CAC-CCC-ATT-GAA-GGT-TCC** | **GGT-TTG-GAA-TGC-TGT-CCA-GGG** |
| ***Gusb*** | NM_010368 | **CTT-CAT-GAC-GAA-CCA-GTC-AC** | **GCA-ATC-CTC-CAG-TAT-CTC-TC** |
| ***Hprt*** | NM_000194 | **TTG-CTG-ACC-TGC-TGG-ATT-AC** | **AGT-TGA-GAG-ATC-ATC-TCC-AC** |
| ***Hsl*** | NM_001039507;NM_010719 | **GTG-TGT-CAG-TGC-CTA-TTC-AG** | **GTC-AGC-TTC-TTC-AAG-GTA-TC** |
| ***Igf1*** | NM_001111276;NM_001111274;NM_184052 | **ACC-AAA-ATG-ACC-GCA-CCT-GC** | **AAC-ACT-CAT-CCA-CAA-TGC-CTG-TC** |
| ***Il1b*** | NM_008361 | **ACT-GTT-CCT-GAA-CTC-AAC-TG** | **CTT-GTT-GAT-GTG-CTG-CTG-CG** |
| ***Il6*** | NM_031168 | **AGT-TGC-CTT-CTT-GGG-ACT-GAT** | **TCC-ACG-ATT-TCC-CAG-AGA-AC** |
| ***Nqo1*** | NM_008706 | **GGC-CGA-TTC-AGA-GTG-GCA-TCC-TG** | **TCT-GCA-TGC-GGG-CAT-CTG-GTG** |
| ***Nr1c1*** | NM_011144; NM_001113418.1 | **AAG-GGC-TTC-TTT-CGG-CGA-AC** | **GTT-CAT-GTT-GAA-GTT-CTT-CAG** |
| ***Nr1c3*** | NM_001127330;NM_011146 | **TCT-CTC-CGT-AAT-GGA-AGA-CC** | **GCA-TTA-TGA-GAC-ATC-CCC-AC** |
| ***Nr1i2*** | NM_010936.3 | **AGG-AGG-AGT-ATG-TGC-TGA-TG** | **CTT-CAG-GAA-CAG-GAA-CCT-GTG** |
| ***Scd1*** | NM_009127 | **GAT-ACA-CTC-TGG-TGC-TCA-AC** | **AAC-GTG-GTG-AAG-TTG-ATG-TG** |
| ***Sepp1*** | NM_009155,3 | **ATG-ACA-GAT-GTG-GCC-GTC-TTG-TGT** | **GCC-TCT-GAG-GGC-TCC-GCA-GT** |
| ***SrebF1*** | NM_011480 | **ACG-GAG-CCA-TGG-ATT-GCA-CA** | **AAG-GGT-GCA-GGT-GTC-ACC-TT** |
| ***Sult1e1*** | NM-023135.2 | **TCT-TGG-CAA-GGC-CAG-ATG-AC** | **TCC-CAA-AAT-GAT-GCT-GGA-AGG** |
| ***Tnfa*** | NM_013693.2 | **CCT-CAC-ACT-CAG-ATC-ATC-TTC** | **TGG-CAC-CAC-TAG-TTG-GTT-GTC** |
| ***Ugt1a1*** | NM_201645.2 | **GCA-TCT-ATC-TCG-CTG-ATG-AG** | **CAG-AGG-CGT-TGA-CAT-AGG** |
